# Quantitative evaluation of inflammation after phacoemulsification surgery: anterior chamber flare and choroidal vascular index

**DOI:** 10.1007/s10384-025-01220-4

**Published:** 2025-06-12

**Authors:** Metehan Simsek, Gulay Yalcınkaya Cakir, Efe Koser, Cigdem Altan, Muhittin Taşkapılı

**Affiliations:** Department of Ophthalmology, Beyoglu Eye Training and Research Hospital, University of Health Sciences, Istanbul, Turkey

**Keywords:** Anterior chamber flare, Choroidal vascular index, Phacoemulsification

## Abstract

**Purpose:**

To evaluate the relationship between phacoparameters and inflammation parameters (anterior chamber flare (ACF), macular and peripapillary choroidal vascular index (mCVI and pCVI)) changes after cataract surgery.

**Study design:**

Prospective study

**Methods:**

This prospective observational study included patients without systemic and ocular diseases who underwent uncomplicated cataract surgery that may have affected intraocular inflammation. Preoperative, postoperative 1st week, 1st, 3rd and 6th months ACF were measured. At the same visits, mCVI and pCVI were calculated. The relationship between phacoparameters and postoperative ACF, mCVI and pCVI values were evaluated by correlation analysis.

**Results:**

Fifty eyes of 50 patients were included in the study. Postoperative 1st week ACF was significantly higher than preoperative ACF (p<0.001). ACF decreased significantly from postoperative 1st week to 6th months. Postoperative 1st month and 3rd month mCVI were significantly higher than preoperative mCVI (p<0.001, p=0.04, respectively). It was observed that pCVI reached its peak value in the 1st postoperative week and decreased to base value in the postoperative 6th month. A strong positive linear correlation was found between total cumulative dissipated energy (CDE) and the difference between postoperative 1st week and preoperative ACF (p<0.001, r=0.68).

**Conclusion:**

Particularly in the early period after phacoemulsification, ACF, mCVI and pCVI increased. The increase in ACF lost its significance at the third month and the increase in mCVI and pCVI remained significant, which may indicate that the inflammatory effect of cataract surgery lasts longer in the posterior segment. Total CDE can be used to predict postoperative inflammation levels.

## Introduction

Cataract, characterized by opacification of the crystalline lens, is a common cause of reversible blindness [1]. Cataracts commonly occur as a result of ageing and oxidative stress. However, many non-modifiable and modifiable risk factors can affect cataract progression [1, 2]. Cataracts generally develop slowly with a gradual decrease in visual acuity. Complaints such as decreased visual acuity, blurred vision and glare may occur in older age [3, 4]. So, cataract surgery is required in patients with visual complaints. The most common cataract surgery technique is phacoemulsification, which uses ultrasound energy to break the cataract into particles small enough to be aspirated through a handpiece [5].

**Figure 1:**
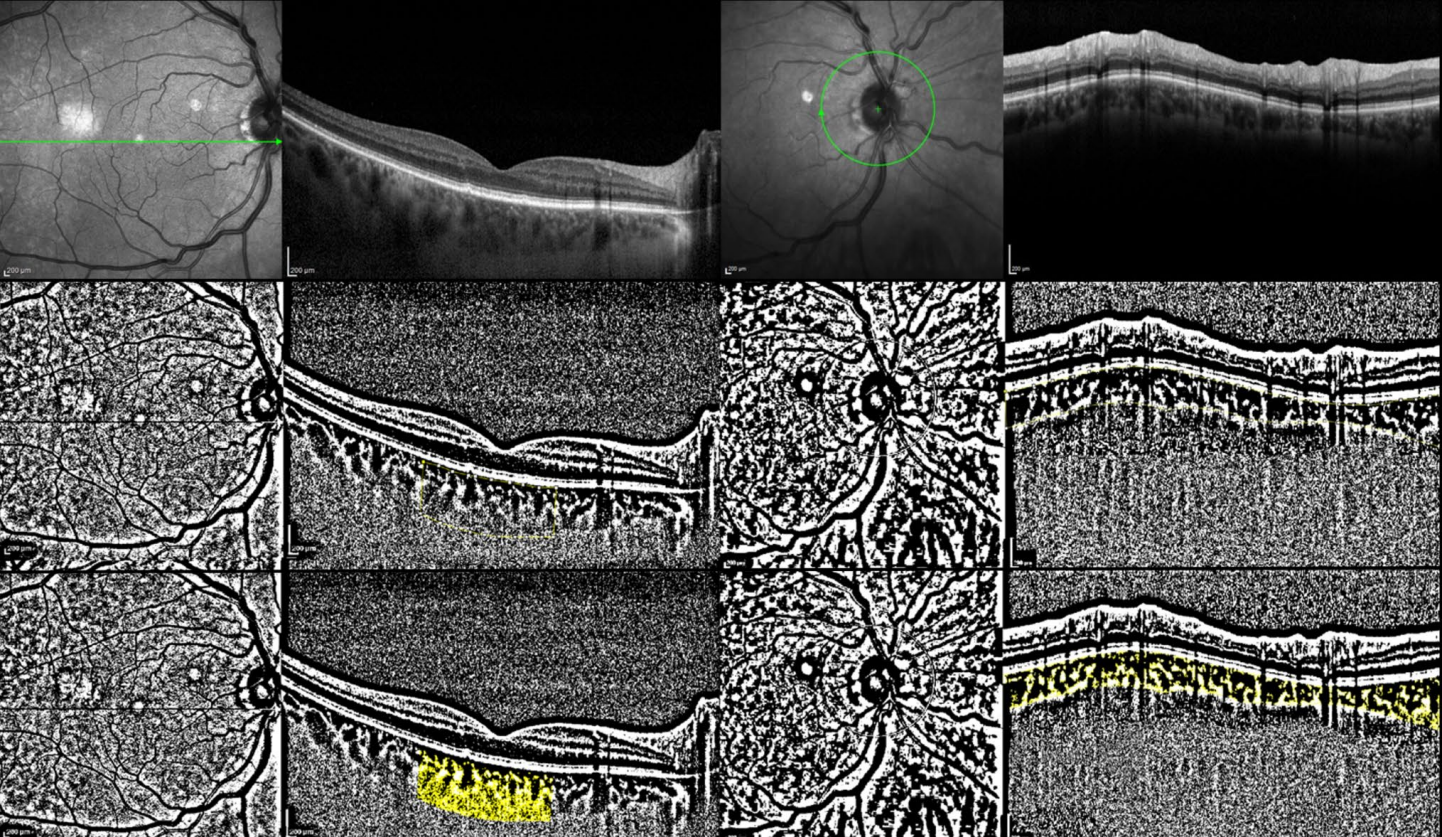
An example of macular and peripapillary choroidal vascular index measurement. Macular enhanced depth imaging optical coherence tomography (upper-left) and retinal nerve fiber layer optical coherence tomography (upper-right) scans, binarized images (middle-left and middle-right). High-light luminal areas using the color thresholding tool (bottom-left and bottom-right).

**Figure 2:**
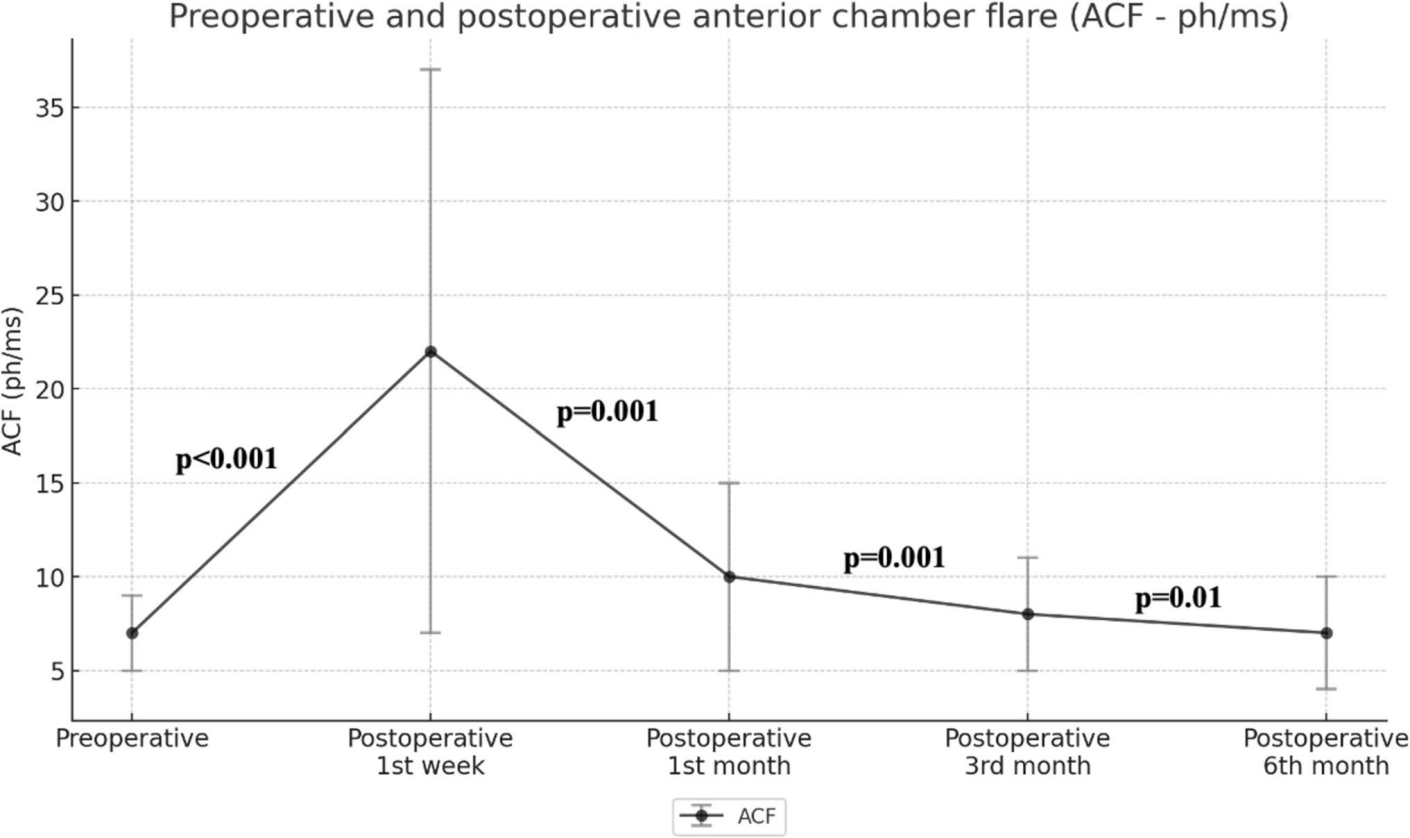
Preoperative and postoperative values of anterior chamber flare is shown as line graph with error bars.

**Figure 3:**
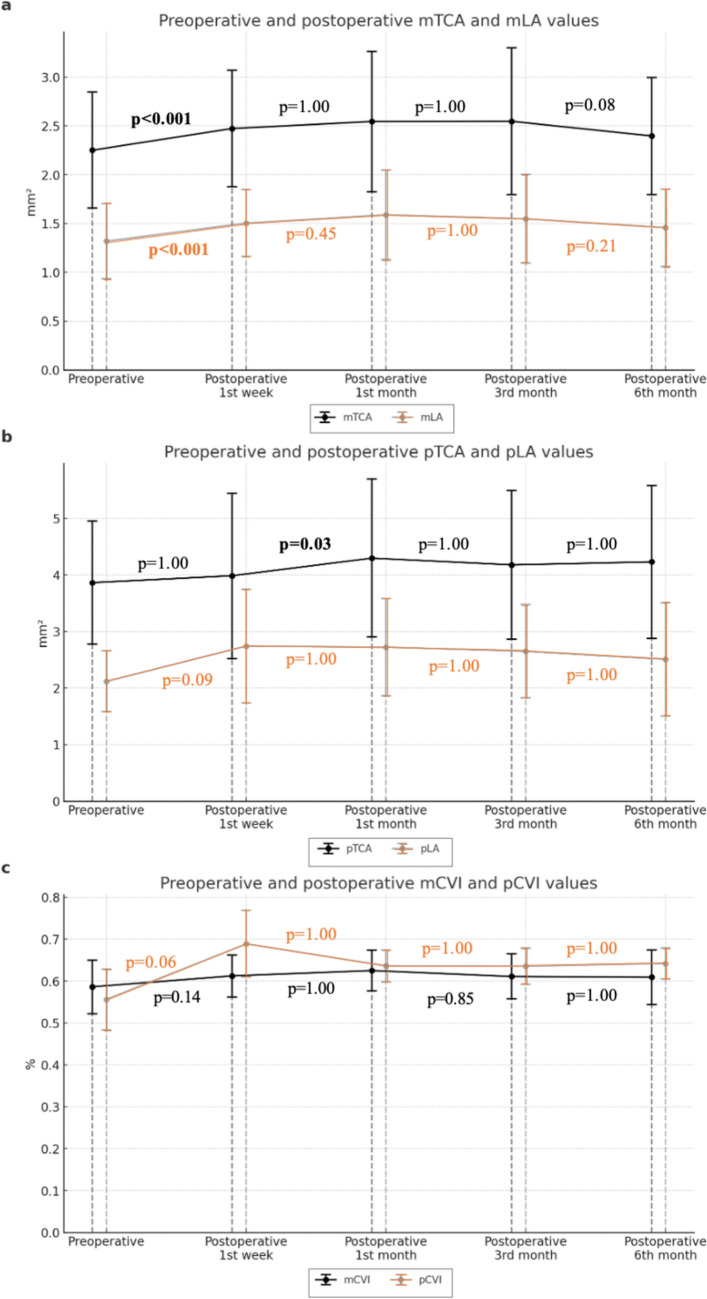
Preoperative and postoperative values of mTCA and mLA (a), pTCA and pLA (b) and mCVI and pCVI (c) are shown as line graphs with error bars (TCA: total choroidal area, LA: luminal area, CVI: choroidal vascular index, m: macular, p: peripapillary).

After phacoemulsification surgery, intraocular inflammation may increase during the early postoperative period [6]. Many factors may affect postoperative inflammation. Diabetes mellitus or intraoperative floppy iris syndrome (IFIS), history of vitrectomy, pseudoexfoliation syndrome (PEXS), all of which increase the number of surgical manipulations, require the use of additional surgical instruments and prolong the duration of surgery, thus these may increase the risk of postoperative inflammation [7]. In addition, ultrasound energy and fluid dynamics used in phacoemulsification surgery may also affect postoperative inflammation. Determination of phacoemulsification parameters that increase postoperative inflammation may enable us to predict the severity of postoperative inflammation and may affect postoperative treatment decisions. Anterior chamber flare (ACF), sub-foveal choroidal thickness (SFCT), central macular thickness (CMT) and choroidal vascular index (CVI) can be used for quantitative assessment of intraocular inflammation [8–11]. Postoperative high ACF and CMT alterations may cause decreased visual acuity, whereas inflammation-induced increases in SFCT and CVI may affect photoreceptor functions [12, 13]. All these changes may adversely affect the postoperative visual prognosis. This study aimed to evaluate changes in previously mentioned inflammation parameters at postoperative follow-up and the relationship between phacoemulsification parameters and inflammation parameters to determine the effects of phacoparameters on postoperative inflammation and visual prognosis (see Figs. 1, 2 and 3).

## Methods

### Participants

This prospective observational study had the approval (decision number 6/8) of the University of Health Sciences Ethics Committee and was conducted according to the tenets of the Declaration of Helsinki. All patients gave informed consent for all procedures applied. Written consent was obtained from all patients prior to surgery. 

Patients who underwent consecutive uncomplicated phacoemulsification surgery with nucleus hardness grade 2 or 3 cataracts were prospectively included [14]. Surgery with complications such as posterior capsular rupture which required intraocular lens (IOL) implantation in the ciliary sulcus was not included. Patients with systemic inflammatory diseases (HLA B-27-related diseases, rheumatoid arthritis, diabetes mellitus etc.), history of uveitis or topical/systemic medication (prostaglandin analogues, steroids or non-steroid anti-inflammatory drugs etc.), intraoperative use of iris hook or capsular tension ring that may affect postoperative ocular inflammation levels were also excluded [15]. Patients with a history of ophthalmic surgery and trauma, concomitant corneal opacities, mature cataracts with an absent red fundus reflex, glaucoma, PEXS, IFIS or history of using α1-adrenergic receptor antagonist drugs that may cause complicated cataract surgery were excluded.

A complete ophthalmic examination via slit lamp biomicroscopy was performed in all patients. For fundus examination, a 90 diopter lens (Volk) was used. Anterior chamber cells were evaluated according to the SUN Grading Scheme [16]. Preoperative and postoperative first week; first, third and sixth month best corrected visual acuity (BCVA) were evaluated with a Snellen chart. Intraocular pressure (IOP) was evaluated via a Goldman applanation tonometer. In all visits, peripapillary and macular zones were evaluated using Spectralis optical coherence tomography (OCT-Heidelberg Engineering) with enhanced depth imaging (EDI) mode (EDI-OCT). CMT and SFCT were also measured. Retinal nerve fiber layer OCT scans were performed using a circular scan pattern centered on the optic disc.

### Measurement of Anterior Chamber Flare

In a healthy eye, slit lamp biomicroscopy shows no inflammatory cells in the anterior chamber. In the presence of inflammation, pro-inflammatory substances and serum proteins migrate into the anterior chamber due to disturbances in the blood-aqueous barrier [17]. Accordingly, the visual characteristics of the anterior chamber change and these changes are subjectively noticeable by slit lamp biomicroscopy. However, objective evaluation of inflammation is more valuable for postoperative follow-up. Measurement of ACF was performed in a darkened room without pharmacological pupil dilation using a laser flare meter (Kowa FM 700, Kowa Company Ltd.) in accordance with the manufacturer's instructions. Kowa FM-700 measures ACF by detecting laser light scattering. A diode laser beam is used in this measurement principle to scan a measuring area, projected into the anterior chamber. At each visit both before and after surgery, seven measurements were performed using a laser flare meter by the same blinded investigator (MS) for all patients under the same conditions. Measurements were performed on the day before surgery and in postoperative first week; first, third and sixth months. The highest and lowest values of the measurements were excluded and the mean and standard deviation of the remaining five values were calculated automatically by the anterior cell flare meter and expressed in photons per millisecond (ph/ms).

### Calculation of choroidal vascular index

As inflammation can significantly affect the vascularity and structural composition of the choroid, CVI can be used as a marker of ocular inflammatory changes. The ratio of the luminal area (LA) to the total choroidal area (TCA) can be used to calculate the CVI [18]. Horizontal choroidal images of the peripapillary and macular zones were obtained at each visit using EDI-OCT. For calculation of CVI, macular and peripapillary EDI-OCT images were binarized with ImageJ 1.51s (National Institutes of Health) using a semi-automated technique [18].

All EDI-OCT scans were performed between 10 and 12 am. Measurements of CVI, LA and SA were analyzed independently by 2 blinded researchers (MS, EK). Only high-quality scans with a signal strength >8 were used for the calculations. Macular and peripapillary EDI-OCT images were visualized in ImageJ to calculate CVI. The drawing tool was used to mark the sub-macular area; a line was drawn from the foveola temporally and nasally for 1500 μm, a total of 3000 μm. Using the Polygon tool, the choroidal area between the RPE and choroidoscleral junction was selected manually as TCA. After converting the scans to 8-bits, auto local thresholding, Niblack was used to binarize the selected area and determine the vascular lumen. Using the color thresholding toolset, luminal regions were highlighted and then each binary image was converted to a red-green-blue (RGB) color image. Then, both areas were combined using the AND command. Subtracting LA which represents the sum of all dark areas from TCA, SA was obtained. The same procedure was used to evaluate the peripapillary CVI (pCVI) in the retinal nerve fiber layer OCT scans (Fig. 1). The mean macular CVI (mCVI) and pCVI values were statistically analyzed. To evaluate the intra- and inter-observer reliability of CVI measurements, participants' intraclass correlation coefficients (ICC) with 95% CI were used [19]. For this purpose, the ICC was evaluated using 50 images selected randomly. For mCVI and pCVI, ICC values were 0.92 and 0.94, respectively, indicating high reproducibility.

### Surgical technique

All surgery was performed by the same experienced surgeon (CA). A side port incision was made using a microvitreoretinal blade. An ophthalmic viscous surgical device (OVD) was inserted to the anterior chamber. The temporal side was then incised with a clear corneal tunnel incision. After using the cystotome to perforate the anterior capsule and create a flap, a continuous curvilinear capsulorhexis followed by hydro-dissection was performed. The lens nucleus was fragmented and aspirated with an ultrasonic phaco handpiece using the stop-and-chop technique developed by Paul S. Koch [20]. Meanwhile, balanced saline was used to maintain anterior chamber volume. Cortical lens material was removed using a coaxial irrigation/aspiration handpiece. A one-piece foldable acrylic posterior chamber IOL (Zaraccom) with OVD was placed in the cartridge and implantation in the bag was performed. The OVD was thoroughly cleaned and the incisions were closed with stromal hydration, followed by leak tightness control.

Postoperatively, all patients were administered topical 1% prednisolone acetate 6 times, moxifloxacin 5 times and nepafenac 0.3% once daily. Moxifloxacin was administered 3 times daily and discontinued at the 2nd week. Nepafenac 0.3% was continued at the same dose and discontinued at the 2nd week. Prednisolone acetate was administered 4 times a day in the 2nd week, 2 times a day in the 3rd week and 1 time a day in the 4th week and discontinued at the end of the 4th week.

### Phacoparameters

In all operations, the same phacoemulsification device (Centurion System, Alcon Laboratories, Inc.) was used. At the end of the surgery, each of the following phacoparameters was documented on the device screen: Overall cases (total case time-min, total U/S time-min, total cumulative dissipated energy (CDE - percent-sec), total aspiration time-min, total estimated fluid aspirated-cc) and ultrasonic details (average torsional amplitude (%), average torsional amplitude in foot-pedal position 3 (FP3 - %), total torsional amplitude on time-min, equivalent average torsional amplitude (FP3 - %), average longitudinal power (%), average longitudinal power (FP3 - %), total longitudinal power on time-min, equivalent average ultrasonic power (FP3 - %)).Calculation of phacoparameters was as in the study of Cakir et al. [21].

### Statistical analysis

For statistical analysis, SPSS v26 was used. For the power analysis, an alpha level of 0.05 with 95% power with a sample size of 39 is recommended. To evaluate the distribution of the data, Shapiro-Wilk test, skewness and kurtosis were used [22]. Numerical variables are expressed as means and standard deviations (SD), categorical variables are expressed as numbers and percentages. For statistical analysis, BCVA was transformed from the Snellen chart to the logarithm of the minimum angle of resolution (logMAR) chart. Repeated measures analysis of variance (ANOVA) was performed for comparisons. When combining more than two groups, pairwise comparisons were made using the Bonferroni correction to reduce type 1 errors. A p-value of less than 0.05 was considered statistically significant. Correlation analysis was performed with Pearson's test. Correlation grading (*r* value) was set at 0 - 0.19 very weak, 0.20 - 0.39 weak, 0.40 - 0.59 moderate, 0.60 - 0.79 strong and 0.80 - 1.00 very strong.

## Results

Fifty eyes of 50 patients (35 women,15 men) were included in this study. Demographic characteristics and inflammation parameters are shown in Table 1.

**Table 1:** Demographic characteristics and inflammation parameters in cases undergoing cataract surgery at preoperative and postoperative follow-up.

**Eyes undergoing cataract surgery (*n*=50)**			
**BCVA (LogMAR)** **IOP (mmHg)** **Inflammation** **Parameters** -ACF (ph/ms)	**Preoperative** 0.57 ± 1.31 14.24 ± 2.31 7.13 ± 2.61	**Postoperative Postoperative Postoperative Postoperative** **1**^**st**^** week 1**^**st**^** month 3**^**rd**^** month 6**^**th**^** month** 0.12 ± 0.13 0.08 ± 0.07 0.06 ± 0.06 0.04 ± 0.05 18.18 ± 2.15 16.52 ± 2.05 14.20 ± 2.55 14.26 ± 2.31 21.48 ± 18.55 10.41 ± 4.19 7.94 ± 2.41 6.96 ± 2.08	***p*-value** <0.001* <0.001* <0.001*
-CMT (µm)	228 ± 29	227 ± 30 226 ± 33 221 ± 24 212 ± 25	0.14*
-SFCT (µm) -mTCA (mm^2^)	267 ± 82 2.25 ± 0.59	269 ± 78 276 ± 74 277 ± 80 260 ± 72 2.47 ± 0.59 2.54 ± 0.71 2.54 ± 0.75 2.39 ± 0.60	0.12* <0.001*
-mLA (mm^2^) -mCVI -pTCA -pLA (mm^2^) -pCVI	1.31 ± 0.38 0.58 ± 0.06 3.86 ± 1.08 2.11 ± 0.53 0.55 ± 0.07	1.50 ± 0.34 1.58 ± 0.45 1.54 ± 0.45 1.45 ± 0.39 0.612 ± 0.050 0.62 ± 0.04 0.610 ± 0.053 0.60 ± 0.06 3.98 ± 1.46 4.29 ± 1.39 4.17 ± 1.31 4.22 ± 1.34 2.74 ± 1.00 2.72 ± 0.85 2.64 ± 0.82 2.50 ± 1.00 0.68 ± 0.08 0.63 ± 0.03 0.63 ± 0.04 0.58 ± 0.11	<0.001* 0.003* 0.05* 0.02* <0.001*

The data are shown as the mean ± standard deviation (range).

BCVA Best-corrected visual acuity, IOL Intraocular pressure, ACF Anterior chamber flare, CMT Central macular thickness, SFCT Sub-foveal choroidal thickness, mTCA Macular total choroidal area, mLA Macular luminal area mCVI Macular choroidal vascular index, pTCA Peripapillary total choroidal area, pLA Peripapillary luminal area, pCVI Peripapillary choroidal vascular index

^*^Repeated measures ANOVA with Bonferroni correction

^†^Indicates pairwise comparison between preoperative value and postoperative value.

Bold colored values indicate *p*<0.05.

The mean anterior chamber cell count (ACC) at postoperative 1st week was 1.18±1.08. There was a very strong positive correlation between ACC and ACF at 1st postoperative week (p<0.001 r=0.88). Also, a positive correlation was found between ACC and total torsional amplitude on time (min) at postoperative 1st week (p=0.02 r=0.32). Descement membrane folds were present in 8 eyes at postoperative 1st week and 1st week ACF was significantly higher in these cases (descement membrane fold-positive group: 32.87 ph/ms, descement membrane fold-negative group: 17.48 ph/ms) (p=0.001). No significant difference was found between 1st week mCVI and pCVI in eyes with and without descement membrane folds (p=0.13, p=0.31, respectively). The difference between postoperative 1st week and preoperative IOP was 3.94±2.46 mmHg and ACF was 14.34±18.06 ph/ms, a strong positive linear correlation was found between these two values (*p*<0.001, *r*=0.74). The difference between 1st month and preoperative IOP was 2.28±1.81 mmHg, ACF was 3.28±4.28 ph/ms and a weak positive linear correlation was found between the two values (p<0.001, r=0.74)

Postoperative 1st week ACF was significantly higher than preoperative ACF (p<0.001). ACF decreased significantly from postoperative 1st week to 6th months (between postoperative week 1, month 1 p<0.001; between postoperative 1st month, 3rd month p<0.001; between postoperative 3rd month, 6th month p=0.001) (Fig. 2). No significant difference was found between preoperative ACF and postoperative 3rd month and 6th month ACF (p=0.08, p=0.70, respectively). The increase in ACF lost its significance and returned to the basal level at the postoperative 3rd month (Table 1).

One of the parameters that may indicate posterior segment inflammation, CMT, started to decrease after the 3rd month postoperatively, however, no significant difference was found between preoperative and postoperative visit CMT values (p>0.05). Although SFCT increased until the 3rd postoperative month, reached its peak value and then decreased, similar to CMT, no significant difference was found between preoperative and postoperative follow-up CMT values (p>0.05) (Table 1).

Macular choroidal parameters including mTCA, mLA and mCVI reached peak levels in the 1st postoperative month. Afterwards, although all three values showed a decreasing pattern until the 6th postoperative month, they were significantly higher than the baseline. Of the peripapillary choroidal parameters, pTCA reached its highest value in the first postoperative month, while pLA reached its peak value in the postoperative 1st week. Similar to pLA, pCVI reached its peak value in the 1st postoperative week and then started to decrease (Fig. 3). At the 6th postoperative month, pCVI was similar to the preoperative value (Table 1). In addition, a significant positive linear correlation was found between postoperative 3rd month mCVI and postoperative 1st month pCVI (p=0.001, r=0.50). A significant positive correlation was found between the postoperative 3rd month pCVI value and the phacoparameters average torsional amplitude (%), equivalent average ultrasonic power (FP3) (%), average longitudinal power (%) and average longitudinal power (FP3) (%) values (p=0.03 r=0.32, p=0.006 r=0.41, p=0.002 r=0.46, p=0.03 r=0.32, respectively). No significant correlation was found between total case time (min), total aspiration time (min) and ACF, mCVI and pCVI values (p>0.05) (see Table 2).

**Table 2:** Phacoparameters of patients performed cataract surgery

**Phacoparameters (*N*=50)**	
**Overall cases** *-Total Case Time (min)* *-Total CDE (percent-sec)* *-Total U/S Time (min)* *-Total Aspiration Time (min)* *-Total Estimated Fluid Aspirated (cc)* **Ultrasonic details** *-Average Torsional Amplitude (%)* *-Average Torsional Amplitude (FP3) (%)* *-Total Torsional Amplitude on Time (min)* -*Equivalent Average Torsional Amplitude (FP3) (%)* -*Average Longitudinal Power (%)* *-Average Longitudinal Power (FP3) (%)* *-Total Longitudinal Power on Time (min)*	17.91 ± 7.70 7.68 ± 4,07 0.87 ± 0.47 5.58 ± 1.52 91.89 ± 23.42 27.95 ± 7.31 26.33 ± 6.92 0.84 ± 0.45 10.53 ± 2.76 19.20 ± 11.83 0.41 ± 0.35 1.13 ± 1.00
-*Equivalent Average Ultrasonic Power (FP3) (%)*	10.95 ± 2.92

The data are shown as the mean ± standard deviation (range).

CDE: Cumulative dissipated energy

FP3: Foot pedal 3

Of the phacoparameters, a moderate positive linear correlation was found between total CDE and postoperative 1st week IOP (*p*=0.002 *r*=0.42). A strong positive linear correlation (*p*<0.001 *r*=0.68) was found between total CDE and postoperative 1st week ACF. A moderate positive linear correlation (*p*=0.004 *r*=0.40) was found between total CDE and 1st month ACF. Also, a weak positive linear correlation was found between total CDE and postoperative 1st week mCVI (*p*=0.01 *r*=0.35). A weak positive linear correlation was found between total U/S time and postoperative 1st week ACF and mCVI (*p*=0.01 *r*=0.35, *p*=0.01 *r*=33 respectively).

At postoperative 6th month, mCVI was higher than baseline in 11 eyes and pCVI was higher than baseline in 17 eyes. Logistic regression analysis identified postoperative 1st week high ACF and high total CDE as significant predictors of persistent elevated mCVI (OR 0.970, 95% CI 0.914-1.048, p=0.04; OR 0.32, 95% CI 0.853-1.427, p=0.03, respectively), and 1st week high ACF and long total U/S time as significant predictors of persistent elevated pCVI (OR 0.44, 95% CI 0.687-0.976, p=0.02; OR 0.30, 95% CI 0.657-0.887, p=0.03, respectively).

Linear regression analysis identified postoperative 1st week high ACF, high total CDE and high preoperative mCVI as significant predictors of high postoperative 6th month mCVI (β=0.232, 95% CI 0.552-0.627, p=0.01; β=0.27, 95% CI 0.453-0.562, p=0.04, β=0.176, 95% CI 0.563-0.751, p=0.03, respectively). Also, postoperative 1st week high ACF, high total U/S time and high preoperative pCVI was found as significant predictors of high postoperative 6th month pCVI (β=0.254, 95% CI 0.672-0.858, p=0.04; β=0.35, 95% CI 0.645-0.897, p=0.03; β=0.376, 95% CI 0.755-0.842, p=0.03, respectively).

## Discussion

In this prospective observational study, the relationship between phacoparameters and ocular inflammation parameters after uncomplicated phacoemulsification surgery were evaluated.

For objective evaluation of inflammation in the diagnosis and follow-up of uveitis, ACF provides valuable data [23]. ACF values can also be used to evaluate anterior chamber inflammation after intraocular surgery [24–28]. In this study, postoperative visits showed a significant difference in ACF values. Shah et al. show that ACF reached a peak on postoperative 1st day and then decreased over time [29]. Siriwardena et al. measured ACF value as 10.3±0.4 ph/ms before phacoemulsification and this value was higher than our study (7.13±2.61 ph/ms) [27]. Since the patients included in our study had no additional features affecting preoperative inflammation, the ACF values may have been closer to normal ranges. In addition, the positive correlation between the ACC and ACF and the fact that ACF is also higher in patients with signs of inflammation such as descement membrane fold shows the importance of ACF in the evaluation of ocular inflammation.

Abell et al. found the 1st month ACF values following phacoemulsification surgery as 14.6±10.7 ph/ms [28]. In our study, postoperative 1st month ACF values (10.41±4.19 ph/ms) were lower. In our study, the exclusion of the patients who had cataracts with high hardness score may be the cause of the lower ACF. Previous studies show that postoperative elevated ACF returned to baseline only at postoperative 6th month [20]. De Maria et al. report that even at six months after uncomplicated cataract surgery, ACF was significantly higher than baseline levels [25]. In our study, the return of ACF to baseline at the 3rd month may be due to the inclusion of patients who underwent uncomplicated cataract surgery and the absence of postoperative complications.

There are few studies evaluating the changes in CVI after phacoemulsification surgery [28–30]. Chen et al. evaluated mCVI after cataract surgery in cases without ocular and systemic diseases other than cataract and report a baseline mCVI (0.60±0.05) almost similar to our study (0.58 ± 0.06) that increased significantly in the postoperative period [30]. After cataract surgery, oxidative stress increases, prostaglandin and cytokine levels are elevated in the aqueous humour [31],vasodilatation in the choroidal blood vessels occurs, leading to an increase in CVI. While a significant difference was found between mCVI and pCVI, no significant difference was found in SFCT values. The inflammation-induced dilatation of the vessels in the Haller's layer of the choroid may compress the Sattler's layer and the choriocapillaris, resulting in a normal SFCT. However, postoperative increases in CVI may have been observed because of the dilatation of the choroidal vessels. Since these structural changes in the choroidal vessels may take time to return to normal, the ACF values may have returned to baseline before mCVI. Mackenbrock et al. found no significant correlation between phacoparameters and changes in CMT, similar to our study [32]. In contrast to CVI, there was no significant difference between postoperative CMT values, indicating that the effect of inflammation on the choroid was greater than its effect on the retina.

In our study, ACF reached peak values in the postoperative 1st week and decreased to baseline levels in the 6th month. Additionally, mCVI started to increase at postoperative 1st week and continued to be elevated even at the 6th month. This suggests that anterior and posterior segment inflammation starts at the same time, but posterior segment inflammation continues subclinically for a longer time. After cataract surgery, pseudophakic cystoid macular edema (PCME) may occur due to posterior segment inflammation [33]. Fleissig et al. show that choroidal thickness increased in patients with PCME and then decreased with regression of edema [34]. In our study, preoperative SCFT was similar to all postoperative values, and patients with uncomplicated phacoemulsification surgery without high nucleus hardness score were included. This may be why the patients included in the study did not develop PCME. However, inflammation disrupts the blood-aqueous and blood-retinal barriers, leading to increased vascular permeability. Retinal microcirculation may be impaired and hypoxia may occur. This may result in decreased visual acuity and contrast sensitivity [35]. Topical steroid eyedrops can be used to reduce this inflammation.

To manage postoperative inflammation and minimize the risk of endophthalmitis, topical corticosteroids and antibiotics are administered routinely in the early postoperative period. Moxifloxacin is one of the most commonly used antibiotics due to its bactericidal properties, improved intraocular penetration and broad-spectrum activity [36]. Previous studies indicate that NSAIDs play a role in maintaining intraoperative pupillary dilation, reducing perioperative pain, and lowering the incidence of postoperative cystoid macular edema [37]. In our study, the postoperative treatment regimen was similar to the studies in the literature [38, 39]. This is due to the inclusion of patients with uncomplicated phacoemulsification surgery and no systemic disease affecting ocular inflammation. However, in the presence of inflammatory diseases such as diabetes mellitus [40] or complicated cataract surgery, a more extensive anti-inflammatory treatment regimen may be required.

Previous studies show that high total CDE is equivalent to a longer surgical and recovery time due to more energy dissipation within the eye; lower total CDE may be associated with a successful phacoemulsification, more efficient surgery and better outcomes [41]. In our study, total CDE showed a positive linear correlation with the difference between postoperative 1st week and preoperative ACF, early postoperative ACF and mCVI. This may indicate that total CDE can be used to predict postoperative intraocular inflammation. Also in our study, total U/S time was positively correlated with postoperative ACF and mCVI, indicating that motion and heat energy increased postoperative intraocular inflammation.

In the presence of anterior chamber inflammation, aqueous humour drainage is impaired due to trabecular meshwork dysfunction and IOP increases [42]. Similar to this mechanism, in our study, a significant positive correlation was found between the differences in postoperative 1st week and preoperative ACF and IOP values. However, none of the patients had early postoperative high IOP values or IOP spikes to require topical or systemic anti-glaucomatous medications.

The limitations of our study are that topical corticosteroids and nonsteroidal anti-inflammatory drugs affect the results due to their effect on inflammation, even if all patients used the same eyedrops for the same duration. In addition, the small patient group and relatively short follow-up period may also be considered as limitations. Further studies on the effects of different postoperative anti-inflammatory treatment protocols on ocular inflammation or the evaluation of inflammation in patients undergoing complicated cataract surgery are recommended.

In conclusion, after uncomplicated phacoemulsification surgery, ACF peaked in the early postoperative period and reached the baseline level only at postoperative 6th month but CVI started to increase later than ACF and remained elevated for a long time. This study suggests that the follow-up of ACF and CVI may be clinically important in indicating the severity of postoperative inflammation and individualizing anti-inflammatory treatment. In addition, total CDE and total U/S time were positively correlated with ACF and CVI, indicating that these phacoparameters can be used to predict the severity of postoperative inflammation.
